# The Diagnosis and Prognosis of Impingement Syndrome in the Shoulder with Using Quantitative SPECT Assessment: A Prospective Study of 73 Patients and 24 Volunteers

**DOI:** 10.4055/cios.2009.1.4.194

**Published:** 2009-11-25

**Authors:** Jin-Young Park, Seok-Gun Park, Jung-Sup Keum, Jung-Hwan Oh, Joon-Suk Park

**Affiliations:** Department of Orthopedic Surgery, Konkuk University School of Medicine, Seoul, Korea.; * Department of Nuclear Medicine, Dankook University College of Medicine, Cheonan, Korea.

**Keywords:** Shoulder impingement syndrome, SPECT, Diagnosis, Prognosis

## Abstract

**Background:**

Diagnosing impingement syndrome without rotator cuff tear usually depends on the physical examination and roentgenography, and obtaining objective evidence for this condition is at best difficult. The purpose of this study was to ascertain whether quantitatively assessing this condition with using single photon emission computerized tomography (SPECT) can diagnose impingement syndrome and predict the postoperative results.

**Methods:**

Before executing arthroscopic or open treatment, SPECT was performed on 73 patients and 24 volunteers and these people were followed up for 2 years. Any increased uptake on SPECT was investigated by using the axial view, which demonstrated the greatest uptake for the acromion, distal clavicle, greater tuberosity, lesser tuberosity and the coracoid process of the operated and non-operated sides.

**Results:**

The patients who were diagnosed as having impingement syndrome with or without rotator cuff tear showed increased uptake on the operative side compared to the non-operated side in the assessed locations. The greater tuberosity of the humerus could be used for quantitative measurement as a postoperative prognostic factor.

**Conclusions:**

The bone SPECT method is useful for making the diagnosis of patients with impingement sydrome, and the results of quantitative assessment at the greater tuberosity can be used for evaluating the prognosis following the operation.

Impingement syndrome is one of the most common syndromes that causes pain in the shoulder joint.^1^,2) A combination of acromioplasty, rotator cuff repair and rehabilitation is an effective treatment with a proven track record^3^-5) as an alternative to the more conservative treatments, in the event that these conservative treatments do not bring relief from symptoms. However, it is difficult to differentiate among impingement syndrome, rotator cuff tear, calcific tendinitis, subacromial bursitis, biceps tendinitis and acromioclavicular arthritis as all of these cause shoulder pain. Also, physical examination and roentgenography, which are the most commonly used methods for making the diagnosis, do not provide enough information to identify impingement syndrome as the cause of a patient's shoulder pain.

As the latest bone scanning method uses single photon emission computerized tomography (SPECT) to obtain images from computed tomography, it has become possible to obtain three-dimensional images of the shoulders. A bone scan, which is an aff ordable diagnostic tool for analyzing shoulder pain, is highly versatile and it provides high-quality medical images. When using SPECT with a bone scan, a phosphoric acid-based combination was found to increase the uptake at the spots where the bone metabolism is enhanced, and this makes it possible to obtain an early diagnosis before impingement syndrome directly affects the shoulder bones.^6^,7) SPECT bone scintigraphy is also more sensitive than planar imaging because it is possible to separate the underlying and overlying distributions of activity into sequential tomographic images.6)

Controversy still surrounds the diagnostic ability of MRI for identifying some subacromial lesions, so MRI may not be the gold standard.^1^-2) So we can speculate about the necessity of a radiologic examination to diag nose subacromial impingement syndrome.

The purpose of this study was to explore the possibility of using SPECT to diagnose impingement syndrome, with or without rotator cuff tear, in order to make the prognostic assessment. We compared the preoperative quantitative SPECT with postoperative clinical results and we evaluated the patients' preoperative status by quantitative assessment.

## METHODS

We used SPECT preoperatively for 73 cases in which surgery was performed by a single surgeon for treating impingement syndrome, with or without rotator cuff tear. In these cases, the patients underwent arthroscopic subacromial decompression and they received, if necessary, rotator cuff repair using either the arthroscopic or open method. There were 31 men and 42 women and the average age of the patients was 54 years (range, 36 to 91 years). The mean duration of symptoms was 31 months (range, 1 month to 20 years). The surgical findings confirmed that impingement syndrome without rotator cuff tear was observed in 29 cases, with partial thickness rotator cuff tear being observed in 24 cases and full thickness rotator cuff tear being observed in 20 cases (Table 1).

We performed SPECT analysis on both shoulder joints of 24 volunteers to allow us to compare the patients with a control group. Those persons who experienced pain or tenderness in the shoulders or limited mobility in their daily activities, and those persons with a medical history of having surgery performed on the shoulder were excluded from the control group of this study. The average age of the control group was 42 years. There were 6 males and 18 females (Table 1).

The shoulder functional score proposed by the American Shoulder and Elbow Society (ASES)8) was used to evaluate the patients. The ASES shoulder scores included an evaluation of shoulder pain and the functional evaluation was performed preoperatively and at the final follow-up after 2 years. The average preoperative pain score measured by the visual analogue scale was 7 (range, 2 to 10) and the average preoperative ASES shoulder score was 30 points (range, 0 to 67 points). At the time of diagnosis, the average preoperative ASES shoulder score for the patients with impingement syndrome and without rotator cuff tear was 34, it was 31 for the cases with partial thickness rotator cuff tear and it was 21 for the cases with full thickness rotator cuff tear (Table 2).

**Fig. 1:**
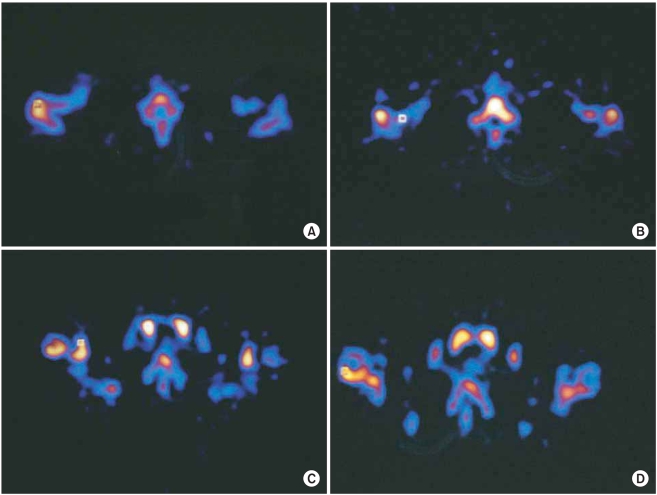
Increased uptake in single photon emission computerized tomography investigated using an axial view with a 1 × 1 square in acromion (A), distal clavicle (B), coracoid process (C), and greater tuberosity (D). The greatest difference between the operative and non-operative side was seen on acromion.

SPECT involved injecting 750MBq of Tc-99m methylene diphosphonate (MDP) intravenously and then obtaining the images at 4 hours after injection. The cameras used for SPECT were the multi-head camera Prism 2000 or the Prism 3000 (Picker®, Cleveland, OH, USA). The tomographic image was reconstructed at 6 mm intervals to capture the sagittal, coronal and axial views. A specialist in nuclear medicine chose the axial images among the three types of images and he reviewed the images as they were projected onto computer screens because the axial images were easier to interpret as compared to the other two types of images. SPECT breaks down the axial images quantitatively into 1 × 1 pixel squares and then it measures the amount of phosphoric acid increase in those squares that show the highest amount of uptake. We measured the painful and contralateral side of the anterior acromion, the distal clavicle, the coracoid process and the greater and lesser tuberosities of the humerus (Fig. 1).

SPECT image was done for all of the 7 patients who complained of pain in both shoulders. For these patients, the operation was performed on the side with the worse symptoms and not for both shoulders. There were 53 operations done on the shoulder of the dominant arm and 20 operations on the shoulder of the non-dominant arm.

To make a quantitative comparison of both shoulders, we employed the paired t-test, and a *p*-value of 0.001 or lower for the paired t-test was considered to have statistical significance. Spearman correlation analysis was done to establish the correlations between the quantitative results of the SPECT analysis and the scores in terms of pain, the functional score and the extent of improvement. A *p*-value of less than 0.05 was considered significant.

## RESULTS

The ASES score was improved to 88 postoperatively (range, 72 to 100). For all the patients of the study group, the quantitative increases of uptake for the bones shown on SPECT were in the positive territory in all sites except for the lesser tuberosity (Table 3). When comparing the ratio between the painful side and the contralateral side (painful side/contralateral side × 100%), the greater tuberosity showed a marked 164% increase in uptake, followed by the distal clavicle and the anterior acromion, which exhibited a nearly 150% increment (Table 3).

The control group did not show a difference in uptake between the dominant arm and the non-dominant arm. We grouped these volunteers into a group with a large amount of quantitative measurement of uptake and another group with a small amount of quantitative measurement of uptake, and then comparing these groups revealed no difference (Table 4). When comparing the patient group to the control group in terms of difference in the SPECT-assisted quantitative measurement, the distal clavicle of the patients group showed a 0.002 *p*-value, while the other parts in the patient group were significantly increased (*p * < 0.001).

According to the intraoperative diagnosis, the patients suffering from impingement syndrome without tear and with partial thickness rotator cuff tear showed an increased tendency of uptake in all areas; however, the extent of the increased uptake was statistically significant in only the greater and lesser tuberosities. Th ose patients with full thickness rotator cuff tear also showed a tendency for increased uptake in all areas; however, the extent of the uptake increase was statistically significant only in the greater tuberosity (Table 3).

One patient, who experienced a reduction of uptake in the greater tuberosity as compared to the contralateral side, suffered from impingement syndrome without rotator cuff tear, which brings about pain to both shoulders. This patient reportedly had symptoms for more than 3 years (painful side/contralateral side × 100% : 58%). There were 3 patients who experienced an uptake at the distal clavicle that was smaller than that on the contralateral side; two of these patients had partial thickness rotator cuff tear (82%, 93%) and the other had full thickness rotator cuff tear (70%). There were four patients who had an uptake in the acromion that was smaller than that on the contralateral side, and one of these patients developed impingement syndrome without rotator cuff tear (90%) for one month and the second, who had symptoms for 6 months, had a bursal side rotator cuff tear (91%). The third patient, who is a 52 year old man, underwent arthroscopic subacromial decompression and distal clavicle resection for impingement syndrome in the non-dominant arm and pain and tenderness on the distal clavicle that developed a month before the operation (60%). The last case was a 41-year old female patient who had impingement syndrome without rotator cuff tear. She underwent arthroscopic subacromial decompression for the shoulder pain she had suffered with for 10 years, and she had shown no improvement even after 8 months of conservative treatment.

For the greater and lesser tuberosities, there was a negative correlation between the quantitative measurement of uptake using SPECT and the preoperative ASES score. The preoperative pain and postoperative ASES scores were unrelated to the quantitative measurement using SPECT. It was found that when the uptake increases quantitatively in the greater tuberosity, the lesser tuberosity, the distal clavicle and the acromion, then the amount of improvement, which was evaluated as the postoperative ASES score minus the preoperative ASES score, increased proportionally (Table 5).

## DISCUSSION

Bone scanning is a commonly used test for making the early diagnosis of disorders in the musculoskeletal system as this modality indicates the dynamic status of the bone metabolism. The conventional two-dimensional planar bone scan has developed into a three dimensional test that allows doctors to examine very fine or multi-layered structures by splitting one overall view into the sagittal, coronal and axial planes with SPECT. SPECT technology has been adapted to diagnosing a wide range of musculoskeletal disease including spondylolysis,^9^,10) spondylolisthesis,10) avascular necrosis,^11^,12) muscle necrosis,13) chronic osteomyelitis,14) sacroilitis,2) and primary bone tumor.15) The applications for bone scanning have been extended as a result of its quantification. As a part of the bone scan technology, research is underway to evaluate the results of arthroplasty and to assess the bone minerals' response to treatment.^16^,17)

Bone scanning in shoulders has been used to detect frozen shoulder,^7^,^18^,19) osteolysis of the distal clavicle,^20^,12) pseudoarthrosis of the acromion,21) os acromiale,22) traction apophysitis of the acromion,23) and rotator cuff disease.^7^,18)

Clunie et al.1) conducted clinical research on 24 patients with shoulder pain. He first grouped them according to their clinical status and then he performed a planar bone scan using Tc-99m MDP. The results showed that 19 patients (79.0%) registered an increased uptake, while roentgenography showed abnormal findings in only 8 patients (33.0%). The bone scan is capable of detecting shoulder disease at an early stage. It had been reported that when Tc-99m MDP was coated on the uptake, there is an increased uptake of the anterior structure of the shoulder, the coracoid process, the acromion and the medial humeral head, without any increment of the posterior aspect of the shoulder, and all this was likely to indicate subacromial disease, while abnormal uptake in the posterior aspect indicated adhesive capsulitis. After the initial report, Tc-99m MDP human immunoglobulin in both shoulders didn't reveal any difference between the symptomatic shoulder and the normal shoulder.24) We analyzed the results of the quantitative SPECT obtained in this study according to the anatomic sites. One patient had a decreased uptake in the greater tuberosity of the painful shoulder, while 13 patients among the 73 patients who participated in this research showed an increase of more than 100% to 120% when compared with the uptake in the contralateral shoulder. The other cases (81%) demonstrated an increase from 120% up to 446%. The uptakes of the acromion were reduced in 4 cases and they were increased by 120% or less in 16 cases. The uptake in 73% of the cases was more than 120% as compared with the contralateral acromion, which is lower than that for the greater tuberosity. When compared with the contralateral side, 75% of the lesser tuberosities, 73% of the distal clavicles and 68% of the coracoid processes increased 120% or more. This result indicated that the greater tuberosity was the most suitable site for employing quantitative SPECT in order to diagnose impingement syndrome (Table 3). But there are the limitations of this study such as performing SPECT at various stage of impingement, and there was no comparison between patients with rotator cuff tear and patients with an intact cuff .

When assessing the prognosis of impingement syndrome with using quantitative SPECT, the higher the uptake was in the greater tuberosity, the lesser tuberosity, the acromion and the distal clavicle, the greater was the obtained functional improvement (the postoperative ASES scores-the preoperative ASES scores). The correlation coefficient was 0.32 between the greater tuberosity and the improvement in the ASES score (*p* = 0.007). The greater tuberosity was found to be the anatomic site that allows doctors to achieve the best assessment of the potential postoperative results before performing an operation (Table 5).

Quantitative SPECT is useful for diagnosing impingement syndrome, with or without rotator cuff tear. The greater tuberosity of the proximal humerus is the most sensitive area among those parts that respond to quantitative SPECT. The greater tuberosity of the humerus can be used for quantitative measurement as a prognostic postoperative factor.

## Figures and Tables

**Table 1:**
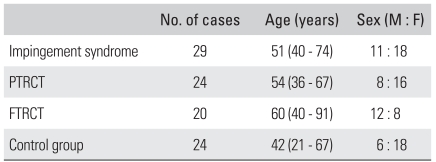
Demographic Findings in Quantitative SPECT Study

PTRCT: Partial thickness rotator cuff tear, FTRCT: Full thickness rotator cuff tear.

**Table 2:**
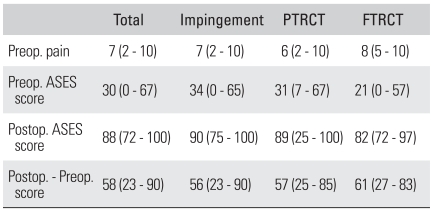
Clinical Data in Quantitative SPECT Study for Impingement Syndrome

PTRCT: Partial thickness rotator cuff tear, FTRCT: Full thickness rotator cuff tear, Preop.: Preoperative, Postop.: Postoperative, ASES: American shoulder and elbow society.

**Table 3:**
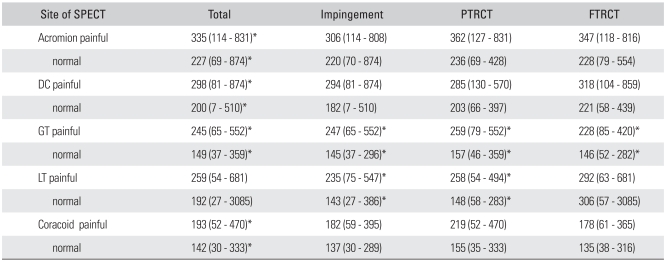
Average Scores and Ranges of Several Anatomic Sites in Painful Shoulder Using Quantitative SPECT

PTRCT: Partial thickness rotator cuff tear, FTRCT: Full thickness rotator cuff tear, DC: Distal clavicle, GT: Greater tuberosity of proximal humerus, LT: Lesser tuberosity of proximal humerus. ^*^p < 0.001.

**Table 4:**
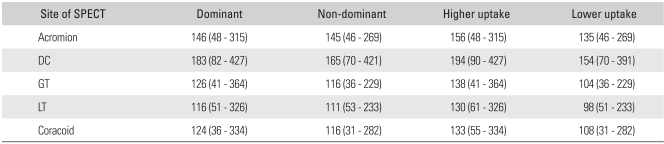
Average Scores and Ranges of Several Anatomic Sites in Volunteer's Shoulder (Control Group) Using Quantitative SPECT

DC: Distal clavicle, GT: Greater tuberosity of proximal humerus, LT: Lesser tuberosity of proximal humerus.

**Table 5:**
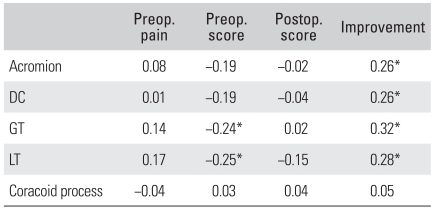
Correlation between Several Aanatomic Sites and Functional Score with Spearman Correlation Scores

Preop.: Preoperative, Postop.: Postoperative, DC: Distal clavicle, GT: Greater tuberosity of proximal humerus, LT: Lesser tuberosity of proximal humerus. ^*^p < 0.05.
